# Annual and state-level data on U.S. total fertility rates, 1931–present

**DOI:** 10.1016/j.dib.2025.111386

**Published:** 2025-02-11

**Authors:** Gregori Galofré-Vilà, María Gómez-León

**Affiliations:** Department of Economic Analysis, Universitat de València, Spain

**Keywords:** Fertility, Births, Baby Boom, Demography, History, United States

## Abstract

This data paper presents a comprehensive reconstruction of U.S. fertility trends, offering state-level Total Fertility Rates (TFR) from 1931 to the present on an annual basis. The dataset computes TFR based on the tabulations of birth counts by maternal age category, sourced from the “Vital Statistics of the United States” published by the U.S. Department of Health and Human Services. Additionally, historical TFR are provided by race, with separate categories for white and non-white populations. TFR were further calculated using the number of women aged 14-49, derived from U.S. census data. This is the first time that TFR is being displayed at the state-year level and captures significant variations in fertility trends across states, during the rapid increase in fertility rates in the 1940s and 1950s, subsequent declines in the mid-1960s, and more recent fluctuations influenced by socio-economic factors, policies, and cultural shifts. By providing detailed, state-level data variation, this new resource offers valuable insights for public health researchers, policymakers, and demographers studying the determinants of fertility trends and their implications for social and economic planning.

Specifications Table

**Table:** 

Subject	Social Sciences
Specific subject area	Annual and State-Level Data on U.S. TFR for white and non-white groups.
Type of data	Panel data.
Data collection	The number of births for white- and non-white groups by maternal age and female population were gathered from the original tabulations of the official statistics of the “Vital Statistics of the United States” (U.S. Department of Health and Human Services). Data were collected using OCR methods, manually checked and inputted into a STATA and CSV files.
Data source location	Primary historical documents using official statistics of the U.S. Department of Health and Human Services.
Data accessibility	Repository name: Mendeley Data identification number: 10.17632/52xszfstsd.1 Direct URL to data: https://data.mendeley.com/datasets/52xszfstsd/1 Instructions for accessing these data: Data available on Mendeley. Published online January 27, 2025.
Related research article	None.

## 1 Value of the Data

• The TFR disaggregated by state- and white- and non-white populations, presents insights on various aspects of the fertility transition in the U.S. (the shift over time from high birth rates to lower birth rates) including the declining birth rates, the delayed childbearing, smaller family sizes, racial differences, and socioeconomic and cultural factors.
• These data are relevant for all social scientists (such as economists, demographers or eco- nomic historians) interested in the regional differences of the demographic transition of the U.S.
• Data may be used to investigate different hypotheses regarding the surge of U.S. fertility during the 1940s and 1950s or the decline in fertility in the mid-1960s.
• The categorization of the TFR by state Inter-university Consortium for Political and Social Research (ICPSR) codes enables flexible linkage with other regional datasets.

## 2 Background

This dataset on state-level TFR from 1931 onward was compiled to support a comprehensive analysis of long-term fertility trends across the U.S. [1]. The primary goal of this dataset is to provide a consistent and thorough resource for examining regional and temporal fertility dynamics, with a particular focus on U.S. demographic transition and the socio-economic factors influencing fertility patterns [2]. The dataset builds upon unused historical data and uses a standardized methodology for calculating TFR, ensuring comparability across time and states [3].

This dataset can be used to investigate how fertility patterns evolved over time and how economic and policy changes during the 20th century influenced them [4]. This period includes significant events in fertility, such as the Baby Boom (a significant increase in the fertility rate occurred between 1946 and 1964, rising from an average of 2 children per woman to almost 4 children per woman) and the Baby Bust (a decline in the fertility rate that began in the mid-1960s, with the fertility rate falling to around 2 children per woman by the 1970s) [5]. To our knowledge, this is the first dataset that harmonizes state-level TFR records over nearly a century, filling a gap in historical and demographic research on fertility trends in U.S. history [1]. While national TFR trends are well-known [6], new TFR data has been compiled at the state level on an annual basis. This work complements the dataset from Bailey et al. (2016) [7], with local crude birth rates. Additionally, the dataset provides insights into both the state-level composition of fertility and its racial components.

This dataset is especially valuable for examining long-term fertility trends and can be linked to state-level determinants using state ICPSR codes, offering insights into regional disparities, temporal shifts, and the socio-economic and policy factors affecting fertility. For example, the *Statistical Abstracts of the United States publish* on a state-yearly basis a wealth of information on economic and social indicators. The panel structure of the data enhances its utility for a variety of research applications, from descriptive analyses to studies aiming to draw causal inferences [5].

## 3 Data Description

**Fig. 1:**
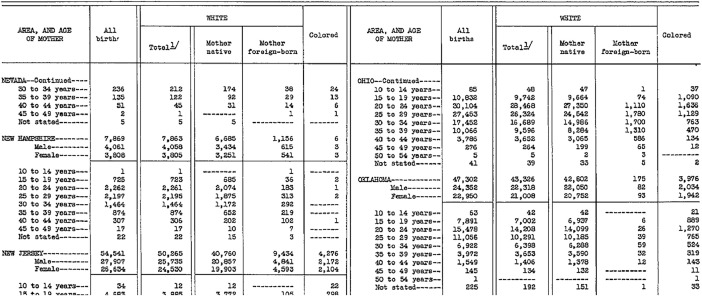
Example of historical records used to compute total fertility rates for U.S. states in 1934, showing the number of births by maternal age groups and distinguishing between white and non-white mothers.

The study utilizes official demographic statistics from the *U.S. Department of Health and Human Services*. For the period 1931–1993, state-level birth data categorized by maternal age were digitized from various volumes of the *Vital Statistics of the United States*. These historical records are publicly accessible via the Centers for Disease Control and Prevention (CDC) website. As an example of the raw historical data, Fig. 1 presents the standard tabulation for 1934. From 1994 to 2020, the data were directly utilized from the CDC's digitized records.

Data transcription was performed using Optical Character Recognition (OCR) analysis through ABBYY software, transcribing the number of births for each state-year, categorized by maternal age and stratified by race. The transcribed data were compiled into an Excel file, where we conducted extensive error-checking to identify and resolve potential transcription errors. This included verifying that the sum of births across maternal age groups matched the totals for each category (total births, white births, and non-white births) and that state-level totals aligned with national figures.

To ensure consistency, we standardized maternal age groupings, as earlier datasets often contained more disaggregated categories. The data were organized into eight age groups: under 15 years, 15–19 years, 20–24 years, 25–29 years, 30–34 years, 35–39 years, 40–44 years, and 45–49 years. Births to mothers over 50 years were excluded due to their rarity.

Using the birth records described above, we calculated the age-specific fertility rate, which represents the number of live births per 1,000 women within specific age brackets. This measure is determined by dividing the number of live births for a given age group by the total population of women in that age group. Age-specific fertility rates were computed for seven 5-year age groups: 15–19, 20–24, 25–29, 30–34, 35–39, 40–44, and 45–49. Population data for females in these age groups were sourced from the U.S. Census and interpolated annually based on the state-level distributions. The sum of all age-specific fertility rates enables the calculation of the total TFR, which represents the average number of children a woman would have if she experienced current age-specific fertility rate throughout her reproductive years. Although data-intensive, the TFR accounts for the age and sex composition of the population, allowing for meaningful comparisons across regions. The dataset, organized in a panel structure, is publicly available in the Mendeley repository.

**Fig. 2:**
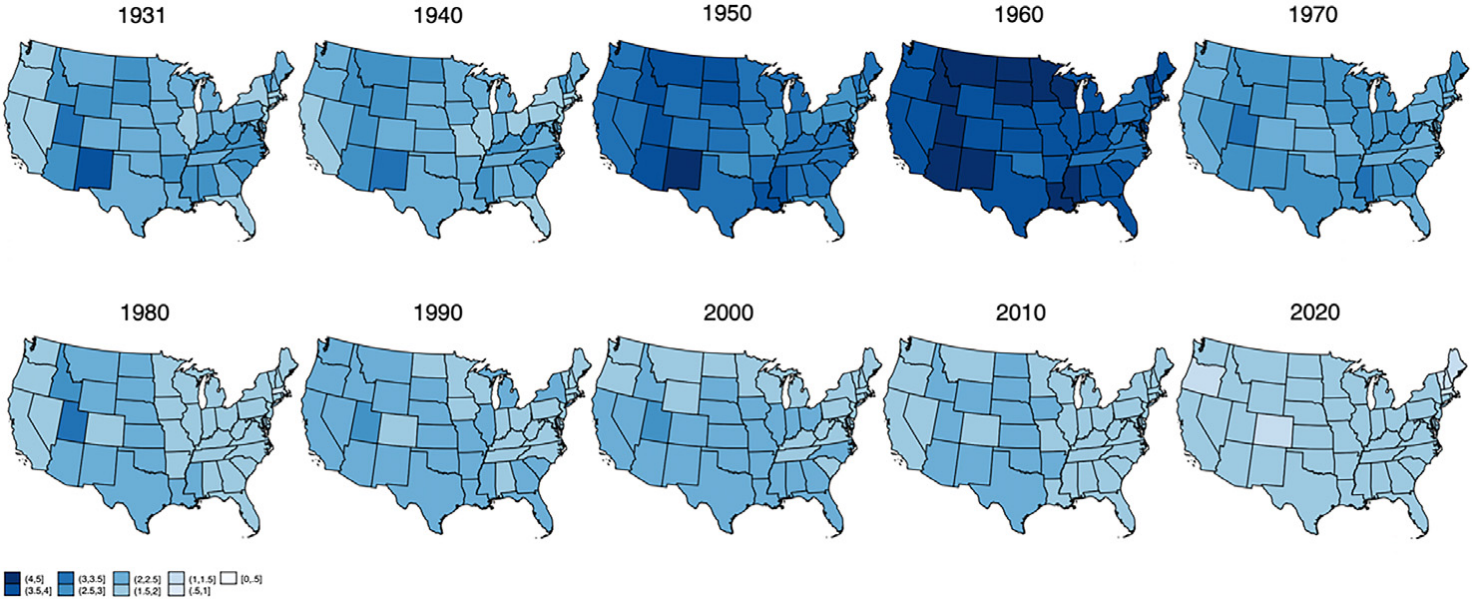
Cross-sectional variation in total fertility rate across U.S. states for selected years (1931–2020), illustrating state-level trends and disparities over time. Own elaboration.

Fig. 2 illustrates the evolution of TFR in different cross-sectional years [1]. Before the 1940s, fertility rates were relatively stable with minor fluctuations. During the 1930s, women in the U.S., on average, had 2.3 children. This rate began to rise in the 1940s, reaching 2.8. However, even in 1931, before the Baby Boom, some states displayed higher TFRs. For example, women in Alabama, Idaho, Kentucky, Virginia, and West Virginia had an average of 2.8 children, while New Mexico, North Dakota, and Utah reported TFRs above 3. Conversely, states like California and Oregon recorded lower TFRs, with women averaging fewer than 1.8 children.

The post-World War II Baby Boom led to a substantial increase in fertility rates, with the TFR peaking at 3.6 during the 1950s. The Baby Boom and subsequent Baby Bust are evident across all states, though there was variation in the peak TFR. In some states, including Arizona, Idaho, Louisiana, Minnesota, Mississippi, Montana, New Mexico, North and South Dakota, Utah, Vermont, Wisconsin, and Wyoming, the average TFR exceeded 4. By 1960, the national TFR was 3.8 and fertility rates began to decline, marking the Baby Bust period, though they remained higher than pre-war levels. By the 1970s, the TFR had decreased to approximately 2, a trend that persisted through the 1980s. After the Baby Boom, states such as Arizona, Idaho, New Mexico, North and South Dakota, Texas, and Utah consistently maintained TFRs above the national average. However, since the early 2000s, fertility rates have steadily declined, reflecting a shift toward smaller family sizes and changes in family planning norms. This downward trend in TFR is evident in nearly all states in recent years.

## 4 Experimental Design, Materials and Methods

The data were transcribed using OCR methods through the ABBYY software, with steps taken to ensure accuracy and consistency. Specifically, we verified that the sum of births across all maternal age groups matched the total number of births recorded for each state. Additionally, we ensured that the aggregate state-level birth totals corresponded to the national figures. In a matrix format, the raw data on fertility has 105,840 cells, including the number births for 49 states, with 90 years of data (starting in 1931 and ending in 2020) and with the births for 7 maternal age groups and a totaling category, for the total, white and non-white populations. In dataset containing the TFR, we have 13,230 cells, with the TFR for 49 states, 90 years of data and the total, white and non-white populations. The variables included in the TFR dataset for each state-year are listed in Table 1, with detailed notes on each variable.

**Table 1:** Content of the related field dataset.

Variable	Variable content
Year	Variable that represents the specific year when the data was collected. It ranges from 1931 to 2020.
State_id	The ICPSR (Inter-university Consortium for Political and Social Research) state codes are a set of numeric codes which uniquely identify geographic areas^1^.
State_name	Variable that represents the name of the state in which the data was collected
Population	Number of women between the ages of 14 and 19. Yearly-state level interpolations are made based on the state-level population from the U.S. Census.
Race	Race of the mother (white and non-white).
TFR	A demographic indicator that estimates the average number of children a woman would have during her lifetime, assuming she experiences the current age-specific fertility rates. It is used to assess population growth or decline and is a key indicator of reproductive behavior. A TFR of 2.1 is considered the replacement level, where a population stabilizes.

## Limitations

Compared to other fertility indicators, such as the crude fertility rate, the TFR offers the advantage of adjusting for the age distribution of mothers, which can be influenced by the local proportion of women in their peak reproductive years. However, the TFR is not without its limitations [8]. A key drawback arises when the timing of childbearing shifts, potentially introducing distortions in the calculations. Additional limitations stem from the use of period measures, often interpreted as “hypothetical” cohorts, and the absence of factors like parity or marriage duration, which may be important for a more comprehensive fertility measure [3]. Nonetheless, despite these shortcomings, the TFR has notable advantages beyond its meaning, particularly its straightforward interpretation compared to other metrics. For example, fertility expressed in births per woman is more intuitive than interpreting whether a general fertility rate of 100 (births per 1,000 women of reproductive age) represents high or low fertility.

Historical fertility data also presents challenges in constructing TFRs. One issue is the presence of a “not stated” category for the age of mothers when recording births. The size of this category depends on the accuracy of data collection across states and varies over time. Nonetheless, this unknown category tends to normalize over time and typically represents a very small fraction of births. For example, as illustrated in Fig. 1 for 1934, the “not stated” category accounted for 22 births in the state of New Hampshire (out of 7,869) and 225 births in the state of Oklahoma (out of 47,302), constituting less than 0.5% in both cases. These minor discrepancies are unlikely to compromise the regional value of the data.

## Ethics Statement

These data were collected without the involvement of human subjects, they were not gathered via animal experiments or social media platforms. No ethical issues are related to these figures.

## CRediT Author Statement

Both authors contributed equally in the Conceptualization, Methodology, Software, Investigation, Validation, Resources, Formal Analysis, Data Curation, Writing, Visualization (Graphs and Maps).

## Data Availability

Mendeley DataAnnual and State-Level Data on U.S. Total Fertility Rates, 1931–Present (Original data)

## Footnotes

1 For details see https://usa.ipums.org/usa/volii/ICPSR.shtml.
